# Phloroglucinol, a nutraceutical for IR‐induced cardiac damage in diabetic rats

**DOI:** 10.1002/ame2.12079

**Published:** 2019-09-12

**Authors:** B. Pranav Nayak, K. R. Ganesha, Nathani Minaz, Rema Razdan, Sumanta Kumar Goswami

**Affiliations:** ^1^ Department of Pharmacology Al‐Ameen College of Pharmacy Bengaluru Karnataka India; ^2^ Department of Pharmaceutical sciences Northeastern University Boston MA USA

**Keywords:** Langendroff apparatus, myocardial reperfusion injury, phloroglucinol, streptozotocin

## Abstract

**Background:**

Myocardial injury due to ischemia‐reperfusion (IR) is aggravated in diabetes which is associated with oxidative stress. Alleviating oxidative stress via use of antioxidants has been shown to be effective at minimizing myocardial cell death and improving cardiac function. The aim of the present study was to evaluate the cardioprotective effect of phloroglucinol against myocardial reperfusion injury (MRI) in diabetic rats.

**Methods:**

Diabetes was induced in female rats with streptozotocin (50 mg/kg). The diabetic rats were orally treated with phloroglucinol (100 and 200 mg/kg daily for 28 days). After treatment the hearts were isolated and mounted on a Langendorff apparatus. The hearts were subjected to 15 minutes of IR to induce myocardial damage. Cardiac functions including heart rate (HR), resting and developed tension, and rate of change of contraction (+d*P*/d*t*_max_) were recorded. Cardiac injury biomarkers lactate dehydrogenase (LDH) and creatine kinase (CK‐MB) were measured in the heart perfusate. Levels of the antioxidant enzymes reduced glutathione (GSH) and malondialdehyde (MDA) were measured. Hematoxylin and eosin (H&E) staining was also performed.

**Results:**

After IR injury, a decrease in HR and +d*P*/d*t*_max_ in hearts from diabetic rat was seen compared to healthy rat hearts, which was reversed by phloroglucinol treatment. Myocardial infarct size, measured by H&E staining, was increased in diabetic rats compared to healthy rats and an increase in the activity of LDH and CK‐MB in the heart perfusate in diabetic rats was decreased by phloroglucinol treatment. An increase in MDA levels and a decrease in levels of antioxidant enzymes were observed in diabetic rats, which was reversed with phloroglucinol treatment.

**Conclusion:**

Phloroglucinol treatment has potential therapeutic promise in the treatment of MRI in diabetes.

## 1 INTRODUCTION

Diabetes is a chronic condition induced in humans by lifestyle changes; once established, it can be controlled to some extent with proper medication.^1^, ^2^ When the condition becomes chronic, it causes complications such as neuropathy, nephropathy, cardiovascular diseases, etc Currently, India is known as the world's “diabetes capital”. In 2017, WHO revealed that India tops the list of countries with the highest number of diabetics, with 31.7 million of over 72 million cases alone that year, and the number is expected to double by 2030.^3^, ^4^

Most patients with coronary artery disease are diabetics. Coronary artery disease, also termed ischemic heart disease (IHD), can be described in simple terms as a reduction in the blood supply to heart muscle due to build‐up of plaque in the arteries of the heart, which later progresses to myocardial infarction.^5^ IHD can also occur due to rupture and dislocation of pre‐existing atherosclerotic plaque when it becomes unstable.^6^

The tissue affected by ischemia undergoes necrosis which is irreversible. Block of the coronary artery leads to hypoxia, which reduces ATP production. This in turn causes an electrolyte imbalance, particularly via Na/K‐ATPase. These changes activate the ischemic cascade finally leading to necrosis and apoptosis of the affected cells.^7^, ^8^

Microvascular injury is a major problem during reperfusion of ischemic heart tissue since there is increased permeability of capillaries and arterioles. During reperfusion, production of reactive oxygen species by endothelial cells results in inflammation. Such reactive species may act indirectly through redox signalling to turn on apoptosis. Further, interleukins released in response to tissue damage may also bind to the endothelium of small capillaries, obstructing them and leading to more ischemia.^9^, ^10^, ^11^

Administration of antioxidants during reperfusion reduces the severity of ischemia‐reperfusion injury. Several substances, including plant extracts, food supplements and even drugs, act as antioxidants, providing one of the therapeutic approaches to overcoming the oxidative stress associated with ischemic heart disease. For example, Banarjee et al demonstrated that chronic garlic administration prevented the oxidative stress and ultrastructural changes associated with IRI.^12^

Phloroglucinol is a benzenetriol, a secondary plant metabolite available for treating GI disorders. It is an algal metabolite and is abundantly found in *Ecklonia cava*.^13^, ^14^ In recent studies it was observed that phloroglucinol has diverse pharmacological activities including antispasmodic activity. Some studies have shown that phloroglucinol has a protective effect against oxidative stress in vitro and in vivo.^15^ Phloroglucinol has also been reported to have antioxidant activity and the ability to decrease the formation of advanced glycation end products.^16^ Furthur, Yoon et al reported that phloroglucinol reduced blood glucose levels in diabetes.^17^ Thus, the present study was conducted to evaluate the protective role of phloroglucinol in myocardial reperfusion injury in diabetic rats.

## 2 MATERIALS AND METHODS

The following reagents were used in the study: streptozotocin/STZ (MP Biomedical Pvt. Ltd), ketamine hydrochloride (Neon Laboratories Limited), xylazine (Indian Immunologicals Limited), CK‐MB kit (Span Diagnostics), LDH kit (Span Diagnostics). Other reagents were of analytical grade.

### 2.1 Animals

Female Wistar rats of appropriate age, weighing 150‐200 g, were used in the study based on availability (64 Female rats). The animals were bred at the animal house of Al‐Ameen College of Pharmacy and reared in large propylene cages in an air‐conditioned room at 24 ± 1°C with a 12 hour light/dark cycle and allowed ad libitum access to water and standard diet. The use of animals for the experiments was approved by the Institutional Animal Ethics Committee (IAEC, reference number: AACP/IAEC/Feb2018/05), and the Committee for the Purpose of Control and Supervision of Experimental Animals (CPCSEA) guidelines were followed.

### 2.2 Experimental design

Type 1 diabetes was induced in overnight fasted rats by intraperitoneal administration of STZ (50 mg/kg body weight dissolved in cold citrate buffer, pH 4.5). A glucose solution (10% w/v) was provided 1 day after STZ injection, and 72 hours after the STZ injection, blood samples were collected after overnight fasting. Animals with fasting blood sugar >250 mg/dL were considered to be diabetic. Non‐diabetic rats were assigned to the normal control group (NC) while rats receiving no treatment were assigned to the diabetic control group (DC). The rats treated with 100 mg/kg phloroglucinol were assigned to the first treatment group (D + Phloro(100)) and rats treated with 200 mg/kg phloroglucinol were assigned to the second treatment group (D + Phloro(200)). The doses were selected based on previous studies.^15^ The treatment was carried out for 28 days.

### 2.3 Heart ex vivo study

At the end of 28 days, blood glucose levels were checked in overnight fasted rats. The rats were then injected with heparin (100 units/rat ip) and after 15 minutes they were anaesthetized with ketamine hydrochloride (65 mg/kg, ip) and xylazine (10 mg/kg, ip). The hearts were isolated and washed in Krebs‐Henseleit (K‐H) solution, mounted on a cannula and perfused with K‐H solution gassed with carbogen gas at 37°C at a constant flow rate of 5 mL/minute using a peristaltic pump (Masterflex Pumps, Cole‐Parmer) in a Langendorff apparatus. A fine thread was tied to the apex of each heart and passed through a pulley to the force transducer (MLT500, AD Instruments) connected to an AD Instrument data acquisition system, and 2 g tension was applied to the heart. The heart was allowed to equilibrate in the thermostatic chamber for 30 minutes. It was then subjected to global no‐flow ischemia for 15 minutes, followed by reperfusion for 30 minutes. Changes in the heart rate, resting tension, developed tension, and d*P*/d*t*_max_ were recorded during reperfusion and compared among the groups.^18^, ^19^

### 2.4 Estimation of cardiac damage

Lactate dehydrogenase (LDH) and creatine kinase (CK‐MB) release was measured to evaluate the presence of cardiac injury. At the end of the experiment, levels of LDH and CK‐MB in the perfusate were determined spectrophotometrically via commercially available LDH and CK‐MB kits.

### 2.5 Biochemical studies

#### 2.5.1 Preparation of heart homogenate

Heart tissue was mixed with 10 times (w/v) ice‐cold phosphate buffer (pH 7.4) and homogenised and centrifuged for 10 minutes at 955.89 *g* at 4°C.

#### 2.5.2 Estimation of lipid peroxidation using the TBARS (MDA) assay

The TBARS or MDA assay is based on the reaction of thiobarbituric acid reactive substances (TBARS) with thiobarbituric acid (TBA) to form a pink colored product. The color intensity at 535 nm or the fluorescence intensity at (*λ*_ex_/em = 530 nm/550 nm) is directly proportional to the TBARS concentration in the sample. TBARS were estimated according to the method of Ohkawa et al.^20^

#### 2.5.3 Estimation of antioxidant enzymes using the reduced glutathione assay

The reduced glutathione assay is based on the glutathione recycling system by DTNB and glutathione reductase. DTNB and glutathione (GSH) react to generate thiobenzoic acid which has a yellow colour. Therefore the GSH concentration can be determined by measuring absorbance at 412 nm. Reduced glutathione was estimated according to the method of Beutler et al.^21^

#### 2.5.4 Determination of myocardial infarct size

Myocardial infarct size is the measure of cardiac remodelling and dysfunction. Infarct sizes were measured according to the method of Jichun Han et al.^22^

#### 2.5.5 Histopathology

Histopathology of the hearts was carried out using H&E (hematoxylin and eosin) staining to observe the changes in cell architecture. Myocardial tissue was fixed in 10% neutral buffered formalin with a tissue:fixative volume ratio of 1:10 for optimal fixation. The tissue solution was routinely processed and embedded in paraffin sections of histology grade. Paraffin sections were cut to thickness of 5 µm, dehydrated with 70%, 90%, and 100% ethanol, infiltrated with paraffin, placed on slides and stained with H&E.

### 2.6 Statistical analysis

All data are expressed as means ± SEM. One‐way analysis of variance (ANOVA) between the groups followed by Dunnet's multiple comparison test was used to assess differences between the groups. Probability values a (=*P* < .001 vs normal control group), b (=*P* < .001 vs diabetic control), and c (=*P* < .01 vs diabetic control) were considered significant.

## 3 RESULTS

### 3.1 Amelioration of haemodynamic parameters by phloroglucinol treatment

**Figure 1:**
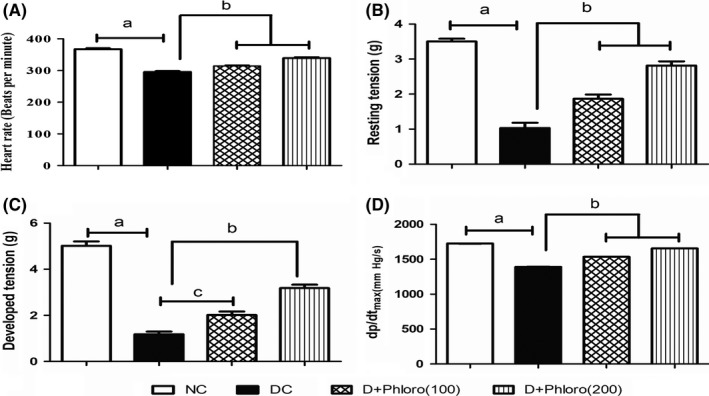
Amelioration of haemodynamic parameters by phloroglucinol treatment. Diabetic rats showed decreases (a) in heart rate (A), resting tension (B), developed tension (C), and d*P*/d*t*_max_ (D) compared to normal rats. Phloroglucinol treatment significantly improved (b, c) the haemodynamic parameters. Values are represented as means ± SEM (n = 6). Significance was assessed by one‐way ANOVA followed by Dunnet's multiple comparison test: a, vs normal control group; b and c, vs diabetic control group. NC, normal control; DC, diabetic control; Phloro(100), 100 mg/kg phloroglucinol; Phloro(200), 200 mg/kg phloroglucinol

The diabetic rats showed a significant decrease in haemodynamic parameters such as heart rate, resting tension developed tension, and d*P*/d*t*_max_ compared to normal rats. Pre‐treatment with phloroglucinol improved the haemodynamic parameters compared to those of diabetic rats (Figure 1).

### 3.2 Phloroglucinol treatment reduces elevated cardiac injury biomarkers

**Figure 2:**
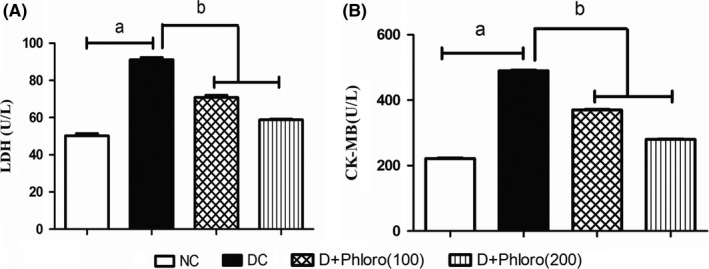
Phloroglucinol treatment reduces elevated cardiac injury biomarkers. Diabetic rats had elevated (a) LDH (A) and CK‐MB (B) levels compared to normal control rats. Phloroglucinol treatment significantly decreased (b) elevated LDH and CK‐MB levels. Values are represented as means ± SEM (n = 6). Significance was assessed by one‐way ANOVA followed by Dunnet's multiple comparison test: a, vs normal control group; b, vs diabetic control group. NC, normal control; DC, diabetic control; Phloro(100), 100 mg/kg phloroglucinol; Phloro(200), 200 mg/kg phloroglucinol

The diabetic rats showed a significant increase in LDH and CK‐MB levels compared to normal rats. Pre‐treatment with phloroglucinol decreased the LDH and CK‐MB levels compared to diabetic rats (Figure 2).

### 3.3 Phloroglucinol treatment enhances antioxidant enzymes and reduces lipid peroxidation

**Figure 3:**
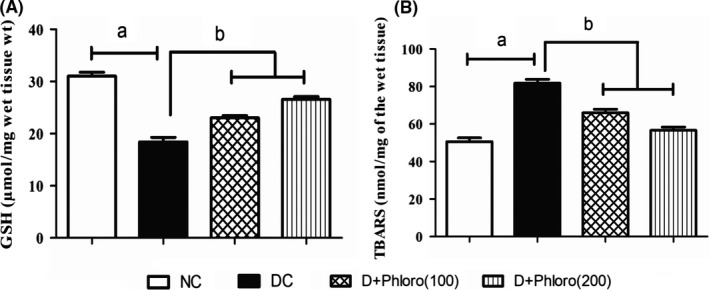
Enhancement of antioxidant enzymes and reduction in lipid peroxidation on phloroglucinol treatment. A, Diabetic rats showed decreased (a) GSH levels and increased (a) levels of TBARS (B) compared to normal rats. Phloroglucinol treatment significantly improved (b) GSH levels and decreased TBARS levels in diabetic rats. Values are represented as means ± SEM (n = 6). Significance was assessed by one‐way ANOVA followed by Dunnet's multiple comparison test: a, vs normal control group; b, vs diabetic control group. NC, normal control; DC, diabetic control; Phloro(100), 100 mg/kg phloroglucinol; Phloro(200), 200 mg/kg phloroglucinol

The diabetic rats had decreased levels of reduced glutathione levels (= antioxidant enzymes) and increased levels of TBARS (MDA = lipid peroxidation) compared to normal rats. Pre‐treatment with phloroglucinol significantly increased reduced glutathione levels and decreased TBARS (MDA) levels compared to that of diabetic rats (Figure 3).

### 3.4 Phloroglucinol treatment alleviates infarct size

**Figure 4:**
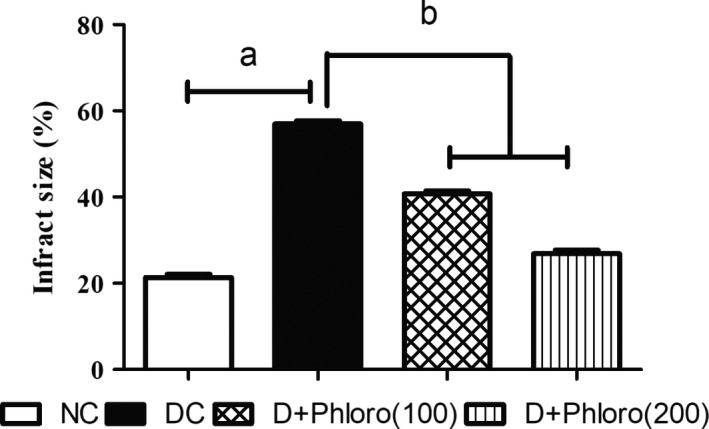
Alleviation of infarct size on phloroglucinol treatment. Diabetic rats showed an increase (a) in infarct size compared to normal rats, which was significantly reduced (b) on phloroglucinol treatment. Values are represented as means ± SEM (n = 6). Significance was assessed by one‐way ANOVA followed by Dunnet's multiple comparison test: a, vs normal control group; b, vs diabetic control group. NC, normal control; DC, diabetic control; Phloro(100), 100 mg/kg phloroglucinol; Phloro(200), 200 mg/kg phloroglucinol

Infarct size in diabetic rats was increased compared to normal rats. Pre‐treatment with phloroglucinol significantly decreased the infarct size compared to diabetic rats (Figure 4).

### 3.5 Phloroglucinol treatment improves the histopathological changes in IR diabetic rat heart

**Figure 5:**
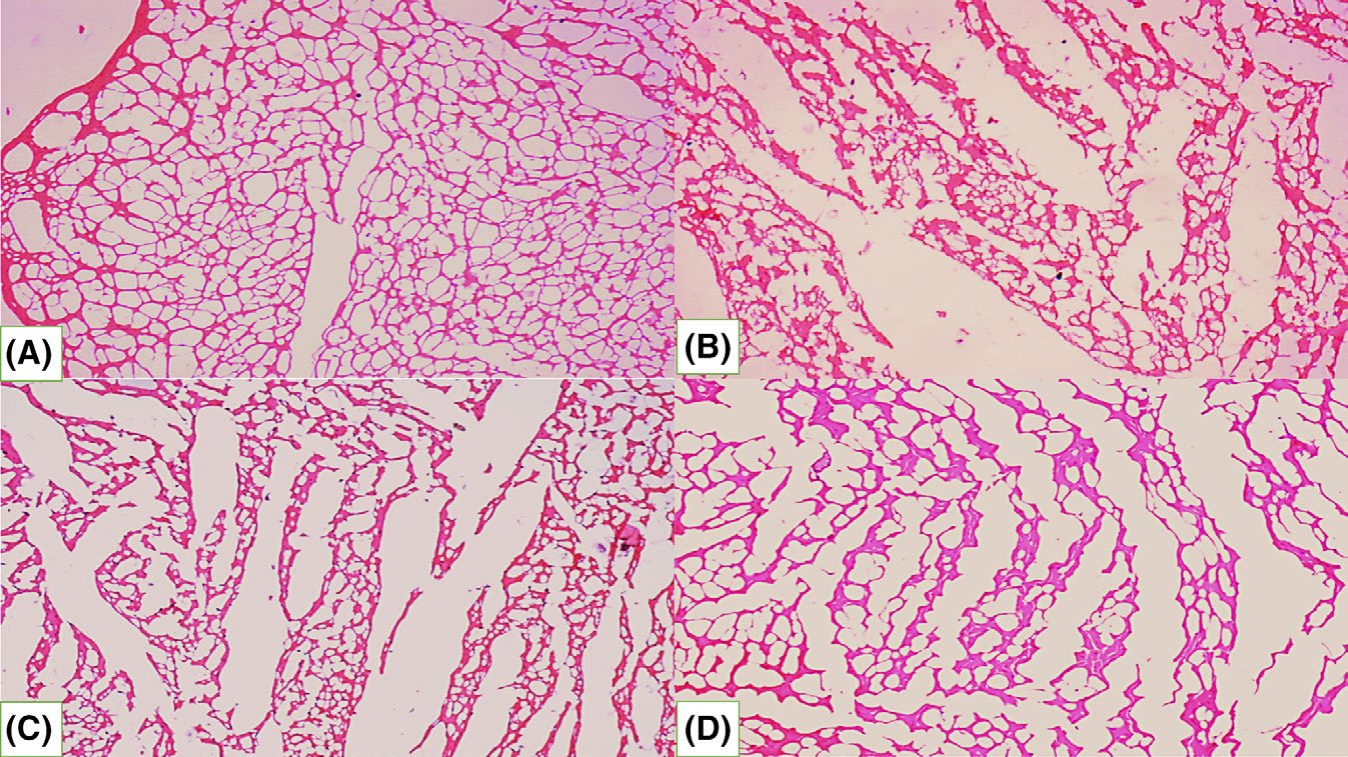
Phloroglucinol treatment improves the histopathological changes in I/R diabetic rat heart. Histopathology of normal (A), diabetic (B), 100 mg/kg phloroglucinol (C), and 200 mg/kg phloroglucinol (D) groups. H&E stain, 10 × magnification

H&E‐stained heart sections revealed that, in the normal group, hearts showed normal structure of the myocardium and areas of haemorrhage were seen (Figure 5A). The hearts of diabetic rats showed myocardial fibre disarrangement and enlarged intercellular spaces (Figure 5B). Treatment with 100 mg/kg phloroglucinol improved the heart structure, which showed occasional loss of muscle fibre and slightly enlarged intercellular spaces (Figure 5C). Hearts treated with 200 mg/kg phloroglucinol showed almost normal structure of the myocardium and only mild histological changes (Figure 5D).

### 3.6 Phloroglucinol treatment alleviates the percentage change in serum glucose seen in diabetic rats

**Figure 6:**
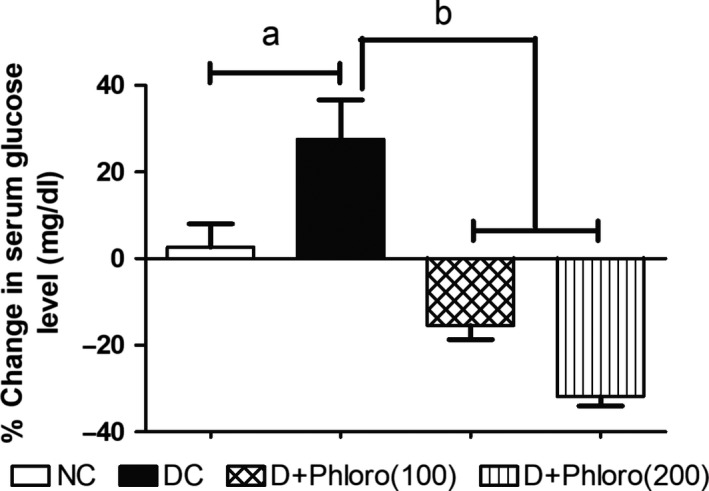
Phloroglucinol treatment alleviates percentage change in serum glucose of diabetic rats. Values are represented as means ± SEM (n = 6). Significance was assessed by one‐way ANOVA followed by Dunnett's multiple comparison test: a, vs normal control group; b, vs diabetic control group. NC, normal control; DC, diabetic control; Phloro(100), 100 mg/kg phloroglucinol; Phloro(200), 200 mg/kg phloroglucinol

Diabetic rats showed a significantly increased percentage change in blood glucose level compared to normal rats. Treatment with phloroglucinol significantly decreased the percentage change in blood glucose levels compared to diabetic rats (Figure 6).

## 4 DISCUSSION

Ischemic heart disease is the result of a partial or complete blockage of the heart's arteries which may be due to atherosclerosis, blood clots, coronary artery spasm, etc. During the reperfusion of the tissue there is an explosion of reactive oxygen species (ROS) and these attack the cells and cause damage. ROS cause cell membrane lipid peroxidation and structural breakdown, and result in leakage of myocardial enzymes. Inhibiting the generation of ROS or the antagonists of reactive oxygen toxicity is important to alleviate myocardial reperfusion injury.^23^ Several studies have reported that phloroglucinol has antioxidant, anti‐inflammatory and antihyperglycemic properties.^15^, ^16^ Therefore, the present study was conducted to evaluate the cardioprotective effect of phloroglucinol against myocardial reperfusion injury in diabetic rats.

Increases in serum glucose levels can lead to structural and functional changes in target organs in diabetic patients. Streptozotocin causes pancreatic β‐cell death and thus reduces the population of these cells. This leads to development of insufficient production of insulin and, consequently, the elevation of blood glucose levels and formation of advanced glycation end products (AGEs). AGEs can cause intracellular changes in vascular and myocardial tissue via interaction with AGE receptors, which leads to cardiac failure.^24^ In our study, the blood glucose levels of STZ‐induced diabetic rats were significantly higher than normal. Treatment with phloroglucinol significantly reduced the blood glucose levels in the diabetic rats, suggesting that phloroglucinol might exert its anti‐hyperglycemic effect by suppressing hepatic glucose production. Phloroglucinol has also been reported to reduce AGE production in diabetic rats,^24^ supporting this hypothesis.

In the present experiments, the heart rate in the diabetic rats decreased compared with controls, in line with the study of Li et al^25^ The decrease in heart rate may be due to an increase in vagal tone or a decline in sympathetic tone, which in turn reduces heart rate.^26^ Treatment with phloroglucinol significantly increased the heart rate compared to diabetic control rats, which suggests that phloroglucinol might have decreased the vagal tone or increased the sympathetic tone of the diabetic rat hearts.

Resting tension and developed tension also decreased in the diabetic rat hearts in our study, in line with the findings of Oliul Islam.^27^ The decrease in tension may be due to stimulation of β_2_ receptors in the heart, resulting in the formation of cAMP through G_s_‐protein. Increased cAMP in vascular smooth muscle inhibits myosin light chain kinase, leading to a reduction in contractile force. Treatment with phloroglucinol significantly increased the resting and developed tension compared with diabetic control rats. This suggests that phloroglucinol might have decreased the stimulation of β_2_ receptors or increased the stimulation of β_1_ receptors in the diabetic rat hearts.

The decrease in d*P*/d*t*_max_ found in the diabetic rat hearts in the present evaluation may be due to activation of the sorbitol pathway, and this was confirmed by cardiac accumulation of fructose. Increased fructose levels lead to the formation of advanced glycation end products, which in turn impair intracellular Ca^2+^ homeostasis, thereby affecting cardiac contractility. Treatment with phloroglucinol significantly enhanced d*P*/d*t*_max_ compared with diabetic control rats, suggesting that phloroglucinol might have reactivated the sorbitol pathway in the treated rats, further slowing down the process of accumulation of fructose and glycation of various intracellular components.^28^

CK‐MB and LDH are biomarkers of cardiac injury and inflammation. These endogenous enzymes are organ specific and leak from the damaged organ during necrosis into the perfusate. LDH and CK‐MB levels were observed to increase in the perfusate after ischemia reperfusion, which shows myocardial damage in the diabetic rats. Treatment with phloroglucinol significantly reduced the LDH and CK‐MB levels, which confirms a cardioprotective effect of phloroglucinol. These results are in accordance with the study of Amani et al,^29^ who reported that HEMADO can provide cardioprotection through opening of mitoK_ATP_ channels in the isolated rat heart.^30^

Downregulation of antioxidant enzymes coexists with oxidative stress in diabetics. A marked increase in TBARS (MDA) levels was observed in the present experiments, which indicated enhanced lipid peroxidation leading to tissue injury. In the present study, treatment with phloroglucinol significantly decreased the MDA levels in heart homogenates from diabetic rats. Our study is in line with that of Sornalakshmi et al,^31^ who demonstrated that ethanolic extract of *Hedyotis leschenaultiana* reduced TBARS levels in the diabetic rat heart.

Glutathione is an endogenous non‐enzymatic antioxidant, which primarily acts as a reducing agent and detoxifies hydrogen peroxide in the presence of the enzyme glutathione peroxidase. Diabetic animals showed depletion of glutathione levels and phloroglucinol treatment prevented the depletion of glutathione levels in heart homogenates. Our results are in accordance with the study of Shafique Ahmada et al,^32^ who demonstrated that beraprost sodium increased GSH levels in celecoxib‐induced cardiotoxicity in rats.

Cytokines and reactive oxygen species mobilize acid hydrolases, which damage myofibrillar proteins, leading to increases in infarct size and eventually to apoptosis or necrosis of the heart tissue.^33^ Phloroglucinol treatment significantly reduced oxidative stress by elevating antioxidant enzyme levels, which might be a possible cause of the reduction of infarct size in treated diabetic rats.

Reperfusion of ischemic tissues releases reactive oxygen species from the endothelial cells, which causes destruction of muscle fibres, infiltration of neutrophils, rupture of cells, necrosis, and haemorrhage, leading to tissue damage. In the present study, myocardial fibre disarrangement and enlarged intercellular space were found the diabetic rats. Treatment with phloroglucinol improved the architecture of the hearts, which showed almost normal myocardial structure, along with mild histopathological changes.

## 5 CONCLUSION

The present study indicates that treatment with phloroglucinol prevents cardiovascular damage in diabetic rat hearts. The treatment was able to protect the heart after ischemia‐reperfusion by preventing haemodynamic changes, preserving antioxidant enzymes, and reversing biochemical and histopathological changes. In summary, our findings support the use of phloroglucinol as a potential therapeutic agent against myocardial reperfusion injury in diabetic rats.

## CONFLICT OF INTEREST

None.

## AUTHOR CONTRIBUTIONS

NM and RR designed the experiments and provided guidance throughout the study. PNB and GKR conducted the experiments. PNB wrote the manuscript. NM, RR and SKG added their opinions and developed and validated the manuscript.
